# The Digital Therapeutic Alliance: Prospects and Considerations

**DOI:** 10.2196/31385

**Published:** 2021-07-20

**Authors:** Reeva Lederman, Simon D'Alfonso

**Affiliations:** 1 School of Computing and Information Systems The University of Melbourne Carlton Australia

**Keywords:** therapeutic alliance, digital therapeutic alliance, digital mental health, mental health apps, teletherapy, chatbots

## Abstract

The growing prevalence of digital approaches to mental health care raises a range of questions and considerations. A notion that has recently emerged is that of the digital therapeutic alliance, prompting consideration of whether and how the concept of therapeutic alliance, which has proven to be a central ingredient of successful traditional psychotherapy, could translate to mental health care via digital technologies. This special issue editorial article outlines the topic of digital therapeutic alliance and introduces the five articles that comprise the special issue.

The therapeutic alliance [1], a measure of the relationship quality between a therapist and their client or patient, is associated with the effectiveness of psychological interventions and successful therapeutic outcomes. Recently, many are turning to online and digital options as a less expensive and more accessible means of therapy than traditional face-to-face care [2]. A growing incidence of mental illness has led to the development of online services by both evidence-based providers operating within health systems and more opportunistic commercial software developers. The need to access help online brought about by COVID-19 can only increase both the demand for online interventions and the desire by developers to meet that demand, which in turn makes the need for online services to prove their efficacy more significant. The increasing prevalence of digital mental health research and interventions has given rise to the term “digital therapeutic alliance” (DTA), which aims to conceptually capture and measure the therapeutic quality of online psychological therapy or digital mental health interventions.

The term DTA is a broad one that can apply to a range of types of digital mental health care. The most straightforward of these systems where the term DTA arises is the alliance between client and therapist in the case of therapy sessions conducted via email, online chat, or videoconferencing. These systems require active input from the therapist despite the intermediary presence of technology to facilitate the interaction. Research suggests that the therapeutic alliance can also be achieved in online modes, such as described above, in the same way that it is in face-to-face therapy and that such digital interventions can have a similar effect as face-to-face therapy [3]. These interactions involve what is known as computer-mediated communication, which is a field of study concerning computing technology use that is relevant to online teletherapy [4].

At the other end of the spectrum of forms of digital mental health care is engagement between a human client and an artificial intelligence (AI)–driven therapy agent. This could range from an online chatbot for mental health [5,6] to robotic or virtual human therapists [7,8]. Such AI-driven therapy agents, from the relatively simple to the more complex, raise a plethora of interesting questions around the nature of the relationship between the human client and the AI therapist. In terms of input from a computing/technology field, human-robot interaction is pertinent [9,10], including questions concerning the psychology of an interaction between a human and an AI agent, particularly its anthropomorphic aspects.

However, most of the work being carried out under the banner of digital mental health concerns web and mobile apps for mental health. Given their prevalence, it is important to determine a conceptualization of therapeutic alliance that users might form with an app and whether this is associated with app effectiveness.

Previous work examining the ability of mental health apps to support therapeutic relationships has stressed the importance of establishing whether the factors that make regular face-to-face therapy effective are the same for digital therapy [11]. If this is the case, how can we incorporate these factors into digital therapies, and if not, what features of an app make it likely to support DTA? Should app developers be trying to recreate face-to-face therapy online, or should the online model have completely different characteristics to traditional therapeutic models? Previous starting points for constructing a conceptualization and measure of DTA include work by Berry et al [12], which adapted the Agnew Relationship Measure (ARM) of therapeutic alliance and developed it into a measure called the mobile Agnew Relationship Measure (mARM). Similarly, Henson et al [13] devised a short DTA measure by selecting 6 items from the Working Alliance Inventory measure of therapeutic alliance and rewording “therapist” to “app.” However, given that such measures are more or less based on existing measures of the traditional therapeutic alliance and simply replace “therapist” with “app,” with possibly a few other minor modifications, ultimately such an approach seems unsatisfactory or incomplete, as it does not account for the possibility of certain nuances, particularities, and complexities that could arise in the context of digital interventions. Furthermore, while there is bound to be some overlap between traditional and digital therapy, one would expect that not all aspects of a traditional therapeutic alliance will necessarily apply to a DTA, and that there may also be dimensions of alliance in the digital context that are not accounted for in traditional therapeutic alliance models.

It is in this context that we invited papers for this special edition on DTA.

The 5 papers published showcase the range of different means through which digital technologies can be used to manage the psychotherapeutic process. They provide an analysis of the current literature in this nascent area and examine the arguments for and against the likelihood of a therapeutic alliance emerging through digital means and how we should view this phenomenon going forward. We need to ask whether the current view of a therapeutic alliance translates well into the digital arena or whether new models should be developed.

In the paper “The Therapeutic Alliance in Digital Mental Health Interventions for Serious Mental Illnesses: Narrative Review” [14], the authors indicate that digital mental health applications offer advantages not found in traditional therapies. These include increased accessibility and autonomy, which can enhance adherence and engagement. They suggest that opportunities for self-guided therapy can lead to unique characteristics for therapeutic alliance in digital contexts. They show that currently the greatest support exists for the effectiveness of digital interventions for anxiety and depression, as opposed to other mental health conditions. They also emphasize the complexity of reaching conclusions in this very diverse field.

In the paper “A Perspective on Client-Psychologist Relationships in Videoconferencing Psychotherapy: Literature Review” [15], the authors emphasize the prescience of this topic during the time of the COVID-19 pandemic. Suddenly, mental health therapy through digital means has become an imperative for many consumers, and so the quality of these therapies in establishing effective treatment has become a more urgent problem to solve. The paper examines DTA in the context of videoconferencing, a close technological option to face-to-face therapy. They suggest conflicting results across their two participant groups, therapists and clients. Clients of psychotherapy were generally satisfied that it was possible to establish a therapeutic alliance through video conferencing. Conversely, therapists expressed concern about the quality of the alliance and the ability to establish a satisfying therapeutic relationship through digital channels. The paper proposes a new model of interaction to deal with these contrasting experiences.

In the paper “Blended Digital and Face-to-Face Care for First-Episode Psychosis Treatment in Young People: Qualitative Study” [16], the authors examined and found client support for a blended care trial intervention, which combined a digital mental health web platform with human moderator support. In this study of young people aged 18-25 years, qualitative data suggest that the blending of online physical and virtual lives in the therapeutic setting was seen as a natural extension of how the clients live the rest of their lives in the current era. Participants in the study identified one of the benefits of blended therapy as strengthening the relationship between the client and the clinician, which is clearly important for DTA. It would be interesting to see this study extended to cover the views of therapists so that it could be compared to the findings of Cataldo et al [15].

In the paper “Impact of Jointly Using an e–Mental Health Resource (Self-Management And Recovery Technology) on Interactions Between Service Users Experiencing Severe Mental Illness and Community Mental Health Workers: Grounded Theory Study” [17], the authors study a scenario where e–mental health resources are available to mental health consumers and workers to use together. In this study, the digital intervention is intended to augment rather than entirely replace face-to-face care, as in a blended system. However, in contrast to an asynchronous system, in this study, mental health workers and clients used the intervention simultaneously during their regular scheduled consultations. The research found that using this form of interaction, relationships were able to be built between mental health workers and consumers. They leave us with a final message, which summarizes the lessons learned from this special edition well: “digital mental health tools should be reframed as tools that can strengthen and augment therapeutic relationships, provided there is a clear shared understanding about how and when they will be used.”

Finally, in the paper “The Digital Therapeutic Alliance and Human-Computer Interaction” [18], the authors start by covering recent nascent work on DTA measures and discussing its limitations, before considering how areas from the field of human-computer interaction (HCI) can play a role in alliance formation and shaping or generating a more suitable, purpose-built measure of DTA. The four areas examined are (1) persuasive system design, (2) affective computing, (3) positive computing, and (4) human-smartphone connection. By exploring the mobile Agnew Relationship Measure of DTA through these HCI lenses, the paper also discusses how HCI methods and knowledge can be used to foster DTA in mental health apps.

We trust that readers will find this special edition interesting, and that it will stimulate future research into the nascent and important topic of DTA.

## Abbreviations

AI: artificial intelligence
ARM: Agnew Relationship Measure
DTA: digital therapeutic alliance
HCI: human-computer interaction
mARM: mobile Agnew Relationship Measure
